# Differential effect of statin use on coagulation markers: an active comparative analysis in the NEO study

**DOI:** 10.1186/s12959-021-00299-2

**Published:** 2021-06-27

**Authors:** Mohammadreza Bordbar, Renée de Mutsert, Melike Cevval, Frits R. Rosendaal, J. Wouter Jukema, Willem M. Lijfering

**Affiliations:** 1 Hematology Research Center, Shiraz University of Medical Sciences, Shiraz, Iran; 2 Department of Clinical Epidemiology, Leiden University Medical Center, Albinusdreef 2, 2333 ZA Leiden, The Netherlands; 3 Department of Cardiology, Leiden University Medical Centre, Leiden, the Netherlands

**Keywords:** Statins, Venous thromboembolism, Coagulation factors, Niacin, Fibrates

## Abstract

**Background:**

Statins are a potential treatment for venous thromboembolism (VTE) prophylaxis complementary to conventional anticoagulants without associated bleeding complications. This study aimed to compare pro-thrombotic activities of different classes of lipid-lowering drugs in an active comparator design and determine whether there is a relation between statin versus fibrate/niacin use and pro-coagulant factor outcomes.

**Methods:**

This is a cross-sectional analysis of participants from the Netherlands Epidemiology of Obesity study using any class of lipid-lowering drugs, including any types of statins, niacin, and fibrates. We performed linear regression analyses to determine fibrinogen, factor (F) VIII, FIX, and FXI activity in statins versus fibrate/niacin users and adjusted for age, sex, tobacco smoking, body mass index (BMI), hypertension, diabetes, and prevalent cardiovascular disease.

**Results:**

Among 1043 participants, the mean age was 58.4 ± 5.2 years, 61% were men, and the mean BMI was 31.3 ± 4.5 kg/m^2^. Clinical characteristics were balanced between statin and fibrate/niacin users. Statin users had lower mean FXI (18.3 IU/dL, 95% confidence interval (CI) 9.4 to 27.3) levels compared to fibrate/niacin users. The level of FVIII (15.8 IU/dL, 95% CI − 0.003 to 31.6), and FIX (11.3 IU/dL, 95% CI − 0.4 to 23.2) were lower in statin users than fibrate/niacin users with marginal statistical significance.

**Conclusion:**

Current statin use was associated with lower plasma levels of FXI than fibrate/niacin use. The effects on coagulation factors may, in part, explain the benefit of statin therapy rendered in primary and secondary prevention of VTE.

## Introduction

Venous thromboembolism (VTE) has an estimated annual incidence rate of 1–2 per 1000 person-years among people of European ancestry [1]. The 3-hydroxy-3-methylglutaryl-coenzyme A (HMG-CoA) reductase inhibitors, the so-called statins, are a class of lipid-lowering drugs widely used to prevent arterial atherosclerotic disease [2].

A growing body of evidence indicates that statins are a promising treatment for VTE prophylaxis complementary to anticoagulants without associated bleeding complications [3–9].

Statins have anti-inflammatory and anti-oxidant properties besides their lipid-lowering effects [10, 11]. Moreover, it has been postulated by mainly in-vitro studies and observational studies that they may have beneficial effects on the vessel wall and anti-thrombotic properties [12, 13]. These include decreased tissue factor expression and thrombin generation, impairment of pro-coagulant reactions catalyzed by thrombin including fibrinogen cleavage and factor (F) V and FXIII activation, reduction of FVII and FVIII activity, enhanced endothelial thrombomodulin expression, and upregulation of fibrinolytic activity manifested by decreased plasminogen activator inhibitor (PAI)-1 and increased tissue plasminogen activator (tPA) expression [14, 15]. In addition, it has also been postulated to have antiplatelet effects by immediate and delayed inhibition of platelet activation, adhesion, and aggregation, although a previous trial could not confirm these findings in vitro [15, 16].

A randomized trial recently showed that 1 month of treatment with rosuvastatin 20 mg/day leads to an improved coagulation profile, most notably decreased FVIII, in patients with prior VTE compared to non-statin users [17]. Given that the effects of drugs are not necessarily class effects, the reduction of pro-coagulant factors by rosuvastatin may not be generalized to other statins currently on the market. It is known that different types of statins show different reducing effects on low-density lipoprotein, atherosclerosis, and inflammation. This reduction is the least strong in pravastatin users, followed by simvastatin and atorvastatin users, and is strongest in rosuvastatin users [16, 18, 19]. A meta-analysis of randomized clinical trials suggested a dose-response relation where rosuvastatin, which is most related to halting or regression of atherosclerosis, dyslipidemia, and inflammation, also provided the most substantial risk reduction in the occurrence of venous thrombosis [20].

We aimed to examine whether there is a relation between statin use and pro-coagulant factor outcomes in individuals participating in the Netherlands Epidemiology of Obesity (NEO) study [21].

## Materials and methods

We performed a cross-sectional analysis of baseline measurements of participants from the NEO study who used a class-specific lipid-lowering drug (statin or fibrate/niacin) and compared their pro-thrombotic activities in an active comparator design.

The NEO study is a cohort study in 6671 individuals aged 45–65 years living in the Leiden area (West of the Netherlands). The majority of the participants have a self-reported body mass index (BMI) of 27 kg/m^2^ or higher. At the baseline visit, blood samples were drawn into tubes containing 0.106 M trisodium citrate (Sarstedt, Numbrecht, Germany) after an overnight fast. Plasma was obtained by centrifugation at 2500×g for 10 min at room temperature and stored in aliquots at − 80° C until testing. Fibrinogen activity was measured according to the method of Clauss. In addition, the activity of FVIII:C, FIX:C, FXI:C, was measured with a mechanical clot detection method on an ACL TOP 700 analyzer (Werfen, Barcelona, Spain). All assays were performed by laboratory technicians who were unaware of the status of the samples.

Since comparisons between statin-users and non-statin users are likely to be confounded, we selected participants who were using any class of lipid-lowering drugs (self-reported), including statins, niacin, and fibrates. The European Society of Cardiology (ESC) and the European Atherosclerosis Society (EAS) guidelines for the management of dyslipidemias recommend that statins are always tried first, and niacin/fibrates are prescribed when statins are not tolerated [22], making niacin/fibrates a better comparator since side effects from statins are expected to be random. In total, 1043 cases were included in the active comparator analysis.

## Statistical analysis

The participants’ general characteristics were reported as means (± standard deviation) or numbers (with percentages). As niacin/fibrates are not associated with anti-thrombotic properties [23], participants who used this class of drugs were regarded as the reference group. The mean of coagulation factors in participants using any statins were compared with the reference group using linear regression and reported as mean difference. The effect size was shown with 95% confidence intervals (CI).

One assumption is that there is no preference in prescribing a lipid-lowering drug to a specific patient and that the clinical characteristics should be distributed evenly over the participants. Given that this assumption might be too strong, we included age, sex, tobacco smoking, BMI, hypertension, diabetes, and prevalent cardiovascular disease (myocardial infarction, angina pectoris, congestive heart failure) as potential confounding factors to the regression analyses. All statistical analyses were computed in SPSS version 22.0.

## Results

The general characteristics of participants (*n* = 1043) who used lipid-lowering medication at baseline are shown in Table 1. The majority of them used five different classes of statins. A small subgroup (*n* = 22) used niacin/fibrates as the lipid-lowering drug. More than two-thirds of the participants reported to smoke (past or current), and nearly half of them were hypertensive (systolic blood pressure (BP) ≥ 140 mmHg and/or diastolic BP ≥ 90 mmHg). About one-third of the patients were diabetic (self-reported diabetes mellitus on medication or fasting plasma glucose > 7 mmol/L) or had impaired fasting glucose (6.1–7 mmol/L).

**Table 1:** General characteristics of the study population

General characteristics	Fibrate /Niacin (*n* = 22)	Fluvastatin (*n* = 10)	Pravastatin (*n* = 114)	Simvastatin ± Ezetimab (*n* = 612)	Atorvastatin (*n* = 181)	Rosuvastatin (*n* = 104)
Mean age, Y (SD)	57.5 ± 4.7	62.3 ± 5.5	58.1 ± 5.2	58.3 ± 5.2	59.0 ± 4.6	57.7 ± 5.5
Men, n (%)	13 (59.1)	4 (40)	71 (62.3)	352 (57.5)	126 (69.6)	72 (69.2)
Mean BMI, kg/m2 (SD)	31.0 ± 2.8	31.8 ± 4.9	31.3 ± 4.5	31.1 ± 4.4	31.4 ± 4.8	31.6 ± 4.7
Smoking history, n (%)	18 (85.7)	8 (80)	86 (75.4)	451 (73.8)	141 (78.3)	86 (82.7)
HTN (Yes), n (%)	10 (45.5)	8 (80)	53 (46.5)	255 (41.7)	88 (48.6)	46 (44.7)
Diabetes (Yes), n (%)	7 (31.8)	6 (60)	39 (34.2)	224 (37)	66 (36.9)	24 (23.5)
CVD (yes), n (%)	3 (13.6)	5 (50)	37 (32.7)	119 (19.5)	77 (43)	39 (38.2)

*BMI* denotes body mass index, *CVD* cardiovascular diseases, *HTN* hypertension

Age and BMI are presented as mean ± standard deviation (SD)

The crude analysis revealed that all coagulation factors were lower in statin users than in fibrate/niacin users except fibrinogen, which was higher in the statin groups (Table 2). The difference was most notable in FXI:C, which showed almost 17 IU/dL lower levels in statin users than fibrate/niacin users (mean difference − 17.1 IU/dL, 95% CI) -30.0 to − 4.3). Adjustment for potential confounding factors did not change the results (mean difference − 18.3 IU/dL, 95% CI − 27.3 to − 9.4). Additionally, current statin users had lower FIX and FVIII (adjusted mean difference − 11.3 IU/dL, 95% CI − 23.2 to 0.4), and − 15.8 IU/dL, 95% CI − 31.6 to 0.003, respectively) with borderline statistical significance. Rosuvastatin users appeared to have lower levels of FVIII and FIX, than users of other types of statins, though these analyses were hampered by small numbers (Table 3).

**Table 2:** Mean difference of coagulation factors among different lipid-lowering drugs users

	Mean level	Mean difference (95% CI)	Adjusted mean difference ^a^ (95% CI)
*Fibrinogen, mg/dL*			
Fibrate/Niacin users (*n* = 21)	294.4 ± 52.5	Reference	Reference
All statin users (*n* = 1007)	316.1 ± 68.4	21.6 (−7.8 to 51.1)	19.1 (−10.0 to 48.2)
Rosuvastatin users (*n* = 104)	316.3 ± 54.0	21.8 (−3.6 to 47.3)	20.7 (−6.2 to 47.6)
Other statin users (*n* = 903)	316.0 ± 69.9	21.6 (−8.5 to 51.7)	18.6 (− 11.0 to 48.4)
*FVIII, IU/dL*			
Fibrate/Niacin users (*n* = 21)	142.1 ± 34.4	Reference	Reference
All statin users (*n* = 1008)	130.5 ± 36.5	−11.5 (− 27.3 to 4.2)	− 15.8 (−31.6 to 0.003)
Rosuvastatin users (*n* = 104)	127.2 ± 33.1	−14.8 (− 30.6 to 0.9)	− 17.6 (− 33.8 to − 1.3)
Other statin users (*n* = 904)	130.9 ± 36.6	−11.5 (− 27.1 to 4.7)	−15.5 (− 31.5 to 0.5)
*FIX, IU/dL*			
Fibrate/Niacin users (*n* = 21)	136.5 ± 29.2	Reference	Reference
All statin users (*n* = 1008)	125.2 ± 27.6	−11.2 (−23.2 to 0.7)	− 11.3 (− 23.2 to 0.4)
Rosuvastatin users (*n* = 104)	122.4 ± 30.5	− 14.1 (− 28.4 to 0.2)	− 14.9 (− 29.7 to − 0.08)
Other statin users (*n* = 904)	125.6 ± 27.3	−10.9 (− 22.7 to 0.9)	− 11.0 (− 22.7 to 0.7)
*FXI, IU/dL*			
Fibrate/Niacin users (*n* = 21)	137.6 ± 28.0	Reference	Reference
All statin users (*n* = 1008)	120.4 ± 20.9	−17.1 (− 30.0 to − 4.3)	−18.3 (− 27.3 to − 9.4)
Rosuvastatin users (*n* = 104)	120.4 ± 18.4	− 17.2 (− 26.8 to −7.5)	− 17.6 (− 27.4 to − 7.7)
Other statin users (*n* = 904)	120.4 ± 21.2	−17.1 (− 26.4 to − 7.9)	− 18.3 (− 27.4 to − 9.2)

*CI* confidence interval

^a^adjusted for age, sex, body mass index, hypertension, smoking, diabetes, cardiovascular diseases

**Table 3:** Comparative results of mean difference in coagulation profile among individuals using different lipid-lowering drugs

	Adjusted mean difference^a^ (95%CI)	Adjusted mean difference ^a^ (95% CI)	Adjusted mean difference ^a^ (95% CI)
*Fibrinogen, mg/dL*			
Fibrate/Niacin users	–	Reference	Reference
Other statin users	Reference	–	18.6 (− 11.0 to 48.4)
Rosuvastatin users	1.5 (−12.1 to 15.3)	–	20.7 (− 6.2 to 47.6)
All statin users	–	19.1(− 10.0 to 48.2)	–
*Factor VIII, IU/dL*			
Fibrate/Niacin users	–	Reference	Reference
Other statin users	Reference	–	− 15.5 (− 31.5 to 0.5)
Rosuvastatin users	−2.6 (− 10.1 to 4.7)	–	− 17.6 (− 33.8 to −1.3)
All statin users	–	− 15.8 (− 31.6 to 0.003)	–
*Factor IX, IU/dL*			
Fibrate/Niacin users	–	Reference	Reference
Other statin users	Reference	–	− 11.0 (− 22.7 to 0.7)
Rosuvastatin users	−2.3 (− 7.9 to 3.1)	–	− 14.9 (− 29.7 to − 0.08)
All statin users	–	−11.3 (− 23.2 to 0.4)	–
*Factor XI, IU/dL*			
Fibrate/Niacin users	–	Reference	Reference
Other statin users	Reference	–	−18.3 (− 27.4 to − 9.2)
Rosuvastatin users	0.3 (−3.8 to 4.5)	–	−17.6 (− 27.4 to − 7.7)
All statin users	–	− 18.3 (− 27.3 to − 9.4)	–

*CI* confidence interval

^a^adjusted for age, sex, body mass index, hypertension, smoking, diabetes, cardiovascular diseases

## Discussion

We found that current users of statins had lower plasma levels of FXI:C than fibrate/niacin users. The results of our study confirm findings of the STAtins Reduce Thrombophilia (START) trial, which concluded that one-month treatment with rosuvastatin 20 mg daily in patients with prior VTE reduced the plasma levels of coagulation factors VII:C, FVIII:C, FXI:C and von Willebrand factor (vWF):Ag in comparison with non-statin users [17]. We also showed that current statin users had 18.3 IU/dL (9.4 to 27.3) lower FXI:C and 15.8 IU/dL (− 0.003 to 31.6) lower FVIII:C than individuals who used fibrates/niacin as their lipid-lowering drugs. The observed difference seems to be mostly related to rosuvastatin use than other types of statins as they consistently showed almost 18 IU/dL lower levels of FVIII:C and FXI:C and about 15 IU/dL less FIX:C compared to non-statin users (Table 3).

The effects of statins on coagulation factor levels were previously noted in different studies. In the Multi-Ethnic Study of Atherosclerosis (MESA) cohort consisting of people free of cardiovascular disease or active cancer, statin users had lower adjusted D-dimer and FVIII levels than non-statin users [14]. Treatment with simvastatin in patients with impaired glucose tolerance and hypercholesterolemia reduced plasma levels of fibrinogen, FX:C, vWF:Ag, PAI-1, and FVII activity. It also led to prolongation of the prothrombin time and activated partial thromboplastin time. Co-administration of ezetimibe with simvastatin showed a synergistic effect on the coagulation profile [24, 25]. Additionally, it was reported that ezetimibe might increase and stabilize the anticoagulant effects of warfarin, particularly when added to statins [26]. Similarly, rosuvastatin but not pitavastatin increased the international normalized ratio (INR) in healthy volunteers on steady-state warfarin [27]. A Dutch study that assessed the immediate and long-term effects of new statin use on the dosage of vitamin K antagonist (VKA) showed that statin users needed lower doses of VKA to achieve the target INR, with the most substantial effect seen with simvastatin and rosuvastatin [28]. Pravastatin was also noted to potentiate the anticoagulant effects of dalteparin [29].

On the other hand, co-administration of rosuvastatin and warfarin in a small number of healthy subjects did not affect warfarin’s steady-state pharmacodynamics [30]. It was also reported that rosuvastatin did not inhibit thromboxane-mediated platelet aggregation in patients with a previous history of VTE [31]. Moreover, one-year treatment with atorvastatin or simvastatin in patients with coronary heart disease had no significant effect on the measured coagulation variables, though they were accompanied by an improved fibrinolytic profile in the treated patients [32]. There have also been reports suggesting that statins do not affect FVII and FVIII levels or activity [33–37].

While the observed differences in coagulation profile, particularly FVIII and FIX, between statin-users and fibrate/niacin-users in our study cohort, were seemingly more attributed to rosuvastatin, we could not detect a difference between rosuvastatin and other types of statin probably because of small numbers. It is believed that the side effects of drugs are not necessarily class effects, especially when the primary mechanism of the drug and the mechanism of side effects are different [38]. Therefore, it makes sense to expect that statins’ anti-thrombotic properties may be limited to some statins. In most observational studies and randomized controlled trials, it was concluded that rosuvastatin use was associated with the strongest (nearly 40%) reduced risk of VTE compared to non-statin users [20, 39–42]. Furthermore, in a Dutch cohort of patients with prior history of pulmonary emboli (PE), highly potent statins with regards to lipid-lowering effects (e.g., rosuvastatin) had the most substantial effect in preventing PE recurrence (hazard ratio (HR) 0.29, 95% CI 0.07–1.16) followed by statins of moderate potency (e.g., atorvastatin; HR 0.44, 95% CI 0.3–0.65) and low potency (e.g., pravastatin; HR 0.88, 95% CI 0.5–1.54) [43]. Despite these findings, several reports emphasize no difference between the type of statin and the risk of first or recurrent VTE [7, 44, 45].

Finally, we showed that contrary to other measured coagulation factors, fibrinogen level was higher in statin users than fibrate/niacin users, though the difference was statistically insignificant. Since fibrinogen is associated with pro-inflammatory and pro-coagulants effects [46], one would expect that statins will decrease fibrinogen levels. A previous study reported that the fibrinogen level reduced after 12 weeks of treatment with simvastatin 20 mg daily in individuals with impaired fasting glucose and hypercholesterolemia [24]. However, in a meta-analysis of 14 other studies, including patients with high serum cholesterol or stable coronary disease, no effect of statins on fibrinogen was found [47]. In a population-based study with 1000 statin users, fibrinogen levels were significantly higher in statin users than in non-users after adjusting for cardiovascular risk factors [14, 47]. We recently confirmed in the START randomized trial [17] that rosuvastatin users had higher fibrinogen levels than non-statin users [48]. It is presently unknown why statins can increase fibrinogen levels, but the timing of plasma fibrinogen level measurement and the type of statin used in different studies may explain such variability.

Our study was conducted with an active comparator design to minimize potential confounding and help us to make treatment groups with similar treatment indications. We compared different statin users with fibrates/niacin users as the reference group. Such a study design was previously explored in the control subjects of the Multiple Environmental and Genetic Assessment of risk factors for venous thrombosis (MEGA) study, which showed statin users had a less hypercoagulable profile than fibrate users. Among the studied statins, rosuvastatin users had the lowest levels of FII, FVII, FVIII, vWF: Ag, FIX, FX, and FXI [38]. However, the latter study was conducted with a small number of participants (*n* = 361), which made the authors warn that the finding should be interpreted cautiously [30]. Therefore, we included a much larger population (*n* = 1043) to overcome this shortcoming and mainly found similar results in MEGA.

Besides, in a cohort of Japanese subjects who were LDL-cholesterol matched at baseline and were treated either with statins or fibrates, the overall mean LDL cholesterol level was lower by 27.3 mg/dl in statin users than in fibrate users (*P* < 0.001) [49]. Furthermore, a meta-analysis of fourteen studies revealed that statin use significantly reduced VTE risk (OR, 0.81; 95% CI, 0.66–0.99, random-effect model), while the use of fibrates was associated with a significant increase in the risk of VTE (OR, 1.58; 95% CI, 1.23–2.02), and niacin did not change the risk of VTE [50]. Thus, it seems that statins are more potent drugs than fibrates in lowering LDL-cholesterol, reducing the risk of atherosclerosis, and reducing coagulation factors, which may ultimately lead to a lower risk of first or recurrent VTE.

Our study was limited by the small numbers of niacin/fibrate users as the reference group. Though we used an active comparator design to minimize confounding, residual confounding might have remained since those prescribed fibrates/niacin may differ from those who received a statin prescription. Besides, we cannot exclude the possibility of adherence bias affecting our results since we included prevalent statins users. Additionally, since the analyses were cross-sectional, causal inferences cannot be made. Setting up a randomized controlled trial is advised to attenuate the possibility of adherence bias, and it is comforting to see that our results are in line with findings from the START trial.

## Conclusion

Current statin use is associated with lower plasma levels of FXI. The type of statin may matter, though it needs further randomized control trials with much larger sample sizes.

## Data Availability

The data used during the current study are available from the corresponding author on reasonable request.

## Abbreviations

VTE: Venous thromboembolism
F: Factor
BMI: Body mass index
CI: Confidence interval
tPA: Tissue plasminogen activator
NEO: The Netherlands Epidemiology of Obesity
ESC: The European Society of Cardiology
EAS: The European Atherosclerosis Society
BP: Blood pressure
START: STAtins Reduce Thrombophilia
vWF: Von Willebrand factor
MESA: Multi-Ethnic Study of Atherosclerosis
INR: International normalized ratio
VKA: Vitamin K antagonist
PE: Pulmonary emboli
HR: Hazard ratio
MEGA: The Multiple Environmental and Genetic Assessment of risk factors for venous thrombosis
